# Suture Versus Non-Suture Closure of the Cystic Duct Orifice During Fenestrating Laparoscopic Subtotal Cholecystectomy: A Single-Center Retrospective Study

**DOI:** 10.3390/jcm15093548

**Published:** 2026-05-06

**Authors:** Hiroto Sakurai, Kei Nakagawa, Shingo Tsujinaka, Kenichiro Yambe, Kazuhiro Takami, Noriko Kondo, Kuniharu Yamamoto, Chikashi Shibata, Yu Katayose

**Affiliations:** 1 Division of Hepato-Biliary and Pancreatic Surgery, Department of Surgery, Tohoku Medical and Pharmaceutical University, Sendai 983-8536, Miyagi, Japan; 2 Division of Gastroenterologic Surgery, Department of Surgery, Tohoku Medical and Pharmaceutical University, Sendai 983-8536, Miyagi, Japan

**Keywords:** cystic duct orifice, fenestrating, laparoscopic subtotal cholecystectomy, reconstituting, suture closure

## Abstract

**Background/Objectives:** Laparoscopic subtotal cholecystectomy (LSC) can be performed using either the fenestrating or reconstituting method. In the fenestrating method, some surgeons additionally perform suture closure of the cystic duct orifice, whereas others do not. However, evidence regarding the clinical significance of suture closure remains limited. **Methods:** Between April 2018 and December 2023, 934 patients underwent cholecystectomy at our institution. Among them, 37 underwent LSC because standard cystic duct control could not be achieved intraoperatively. Of these, 34 were treated with the fenestrating method. Among these 34 patients, 27 did not undergo suture closure of the cystic duct orifice (non-suture group), while 7 underwent suture closure (suture group). Perioperative outcomes were retrospectively compared between the groups. **Results:** No statistically significant differences were observed between the groups in operative time, drain retention period, postoperative hospital stay, postoperative bile leakage, or the need for postoperative endoscopic treatment. Similar findings were observed in exploratory subgroup analyses among patients with intraoperative bile flow from the cystic duct orifice (IBF) and within the non-suture group according to the presence or absence of IBF. No reoperations, readmissions, or deaths occurred in either group. However, postoperative bile leakage (11/27 [40.7%] vs. 1/7 [14.3%]) and endoscopic treatment (7/27 [25.9%] vs. 1/7 [14.3%]) were more frequent in the non-suture group, although not statistically significant. **Conclusions:** In this small retrospective single-institution cohort, no statistically significant differences in perioperative outcomes were observed between patients with or without suture closure of the cystic duct orifice during fenestrating LSC. However, the non-suture group showed a trend toward higher rates of postoperative bile leakage and endoscopic treatment. These hypothesis-generating findings should be interpreted cautiously.

## 1. Introduction

**Figure 1:**
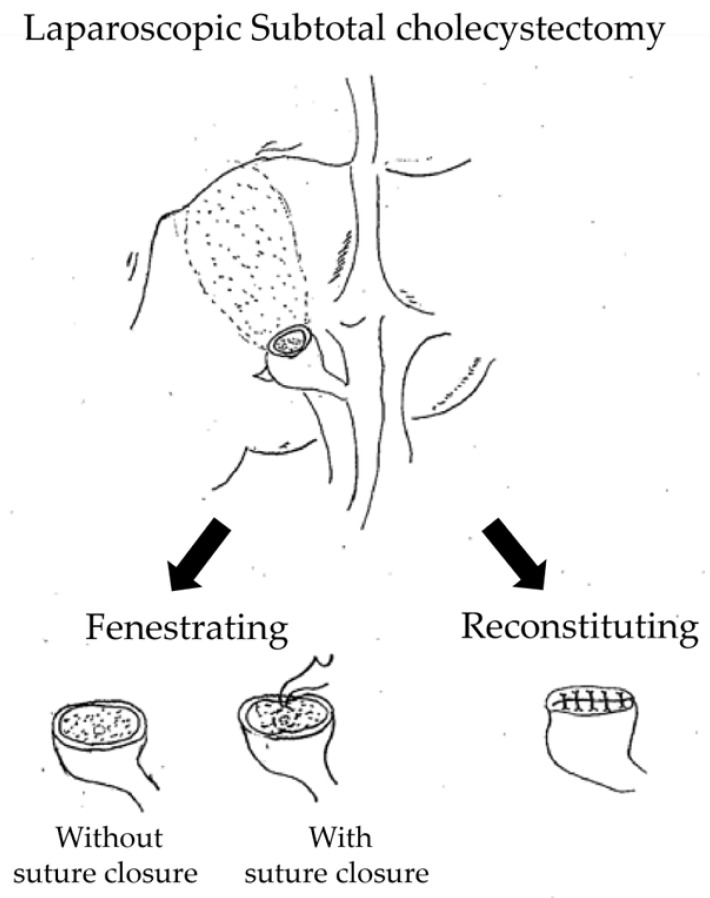
Surgical procedure for laparoscopic subtotal cholecystectomy.

Laparoscopic subtotal cholecystectomy (LSC) is widely accepted as a bailout procedure for difficult cholecystectomy when the Critical View of Safety cannot be achieved and further dissection may increase the risk of bile duct injury [1,2,3,4]. The Tokyo Guidelines 2018 also emphasize the importance of prioritizing safety in such situations [1]. Subtotal cholecystectomy is generally classified into two major subtypes, the fenestrating and the reconstituting methods [3,4]. In the fenestrating method, the remnant gallbladder stump is left open, and the cystic duct orifice may or may not be closed internally with sutures. In contrast, the reconstituting method involves suture closure of the remnant gallbladder stump (Figure 1) [1,3,4,5].

In recent years, comparative studies of fenestrating and reconstituting subtotal cholecystectomy have accumulated, and systematic reviews and meta-analyses have suggested that the reconstituting method is associated with a lower incidence of postoperative bile leakage [2,5,6,7,8]. Simultaneously, this apparent advantage does not fully settle the issue, because remnant-related complications after subtotal cholecystectomy remain a concern, such as retained or recurrent stones, symptomatic remnant gallbladder disease, and remnant cholecystitis requiring further intervention [9,10,11]. For this reason, the technical details of fenestrating LSC still deserve attention, even though the broader comparison between fenestrating and reconstituting techniques has been discussed extensively.

One of the technical questions is whether the cystic duct orifice should be sutured during fenestrating LSC. Internal closure of the cystic duct orifice has been described as a modification of fenestrating subtotal cholecystectomy, and a dedicated suturing technique has also been reported [3,5,12]. A recent large single-institution study suggested that, in fenestrating subtotal cholecystectomy, postoperative bile leakage decreased from 24.1% to 6.0% when cystic duct closure was achieved [13].

Although comparisons between the fenestrating and reconstituting techniques have been increasingly reported [14,15], the impact of suture closure of the cystic duct orifice within the fenestrating method on clinical outcomes has rarely been evaluated. Therefore, this study aimed to evaluate the clinical significance of suture closure versus non-suture closure of the cystic duct orifice during LSC using the fenestrating method.

## 2. Materials and Methods

### 2.1. Patients

**Figure 2:**
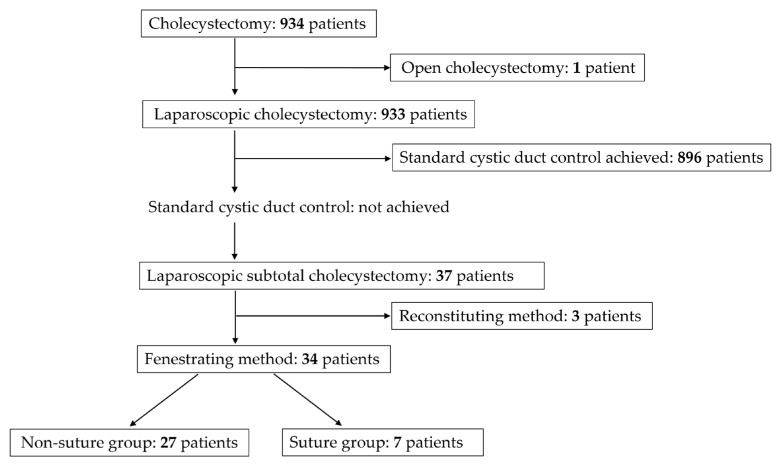
Patient flowchart. Standard cystic duct control was defined as standard clipping or suture ligation of the cystic duct.

Between April 2018 and December 2023, a total of 934 patients underwent cholecystectomy for cholecystitis and gallstone disease at our institution. Among these patients, one patient underwent planned open cholecystectomy, and 933 underwent laparoscopic cholecystectomy, including 8 who required conversion to open surgery. Of the 933 patients who underwent laparoscopic cholecystectomy, 896 underwent standard cystic duct control, defined as clipping or suture ligation of the cystic duct followed by transection. In the remaining 37 patients, standard cystic duct control could not be achieved intraoperatively, and LSC was therefore performed. Of these 37 patients, the fenestrating method was used in 34 and the reconstituting method in 3. The 34 patients treated with the fenestrating method constituted the study cohort and were classified into the suture group (*n* = 7) and the non-suture group (*n* = 27) according to whether the cystic duct was closed with sutures (Figure 2).

### 2.2. Surgical Indications

At our institution, patients with grade III acute cholecystitis are initially managed with gallbladder drainage, such as percutaneous transhepatic gallbladder drainage or endoscopic gallbladder drainage, in accordance with the Tokyo Guidelines 2018 [1]. LSC is not selected instead of non-surgical management but rather is used as a bailout procedure in difficult cholecystectomy cases where the Critical View of Safety cannot be achieved, and bile duct injury is considered likely.

The choice between the fenestrating and reconstituting methods is made intraoperatively. Technically, the reconstituting method may be feasible when the remnant gallbladder stump is not friable and has sufficient length to allow secure closure with sutures. However, closure of the remnant gallbladder stump is often difficult, particularly when the stump cannot be sufficiently detached from the hepatic bed, and may carry a risk of biliary injury. In addition, the reconstituting method may be associated with the risk of residual gallstones, and although rare, remnant gallbladder cancer [2,3,16]. For these reasons, the reconstituting method is not the first choice at our institution and is reserved for highly selected cases.

In patients undergoing the fenestrating method, the decision to perform suture closure of the cystic duct orifice is left to the surgeon’s discretion according to the intraoperative findings. When bile flow is observed from the remnant gallbladder stump, the surgeon may explore the remnant gallbladder cavity to identify the cystic duct orifice and assess whether suture closure is technically feasible and safe. However, in severe acute cholecystitis, the gallbladder wall may be friable or necrotic, the cystic duct may be shortened due to the inflammation, and complex adhesions around the Calot’s triangle may obscure the anatomy. In such situations, suturing the cystic duct orifice may increase the risk of inadvertently involving the common bile duct [3]. In addition, standard port placement for laparoscopic cholecystectomy does not always provide an optimal angle for intracorporeal suturing of the cystic duct orifice. Therefore, when safe suture closure is considered difficult, the cystic duct orifice is left open and abdominal drains are placed (non-suture technique). Accordingly, the decision to perform suture closure was not protocolized and may have reflected intraoperative case complexity.

Completion cholecystectomy after LSC is not routinely considered and is reserved for patients who develop remnant cholecystitis or remnant gallbladder cancer, or in whom a malignant lesion is identified in the resected gallbladder specimen.

### 2.3. Surgical Technique for Laparoscopic Subtotal Cholecystectomy

The standard surgical technique used in our department for laparoscopic subtotal cholecystectomy is described below.

**Figure 3:**
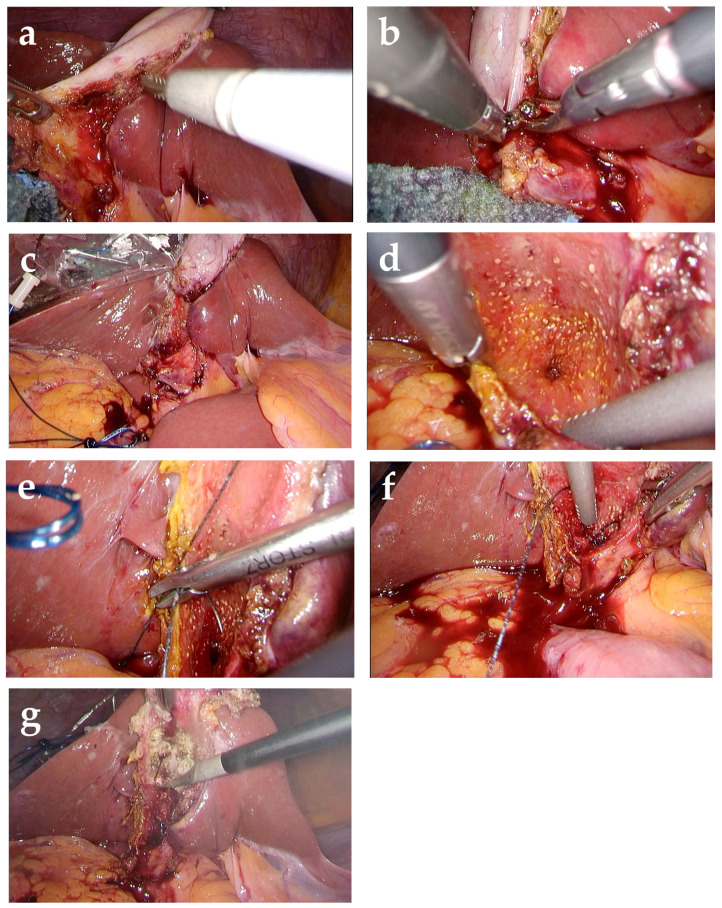
Surgical steps of the fenestrating laparoscopic subtotal cholecystectomy: (**a**) the gallbladder wall is opened and dissected from the neck toward the fundus on the abdominal side; (**b**) gallstones are extracted from the gallbladder lumen; (**c**) the size of the remnant gallbladder is minimized to reduce the risk of stone recurrence; (**d**) the cystic duct orifice is identified within the remnant lumen; (**e**) absorbable sutures are placed at the cystic duct orifice; (**f**) the cystic duct orifice is closed with sutures; (**g**) the mucosa on the hepatic side of the remnant gallbladder is ablated.

**Figure 4:**
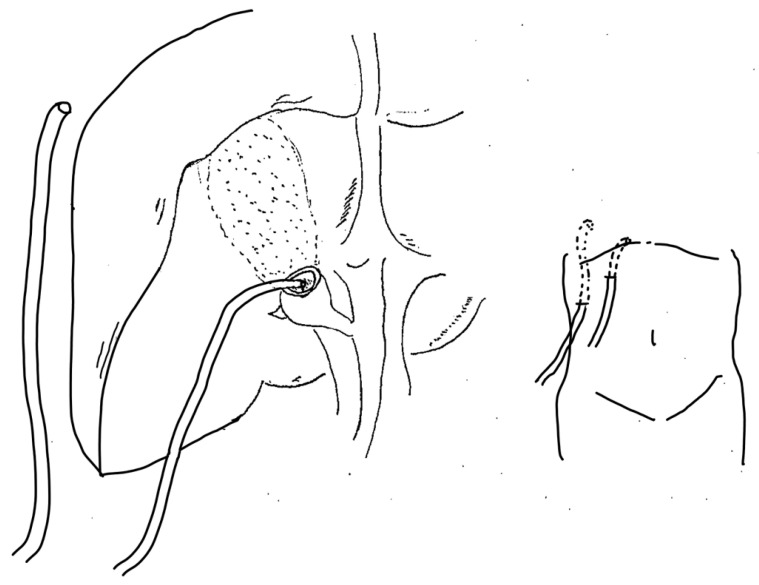
Postoperative gallbladder status and drain placement location.

(1) A 12 mm balloon camera port is inserted through the umbilicus, and the abdomen is insufflated with carbon dioxide to maintain an intra-abdominal pressure of approximately 10 mmHg. Three 5 mm ports are then placed in the epigastrium, right anterior axillary line, and right midclavicular line, enabling the procedure to be performed using a total of four ports. In difficult cases requiring subtotal cholecystectomy, an ultrasonic coagulation and cutting device is used in combination with a hook-type electrosurgical device.
(2) Adhesions around the gallbladder are identified and dissected. If the gallbladder is enlarged and tense, it is punctured and its contents are aspirated.
(3) Rouvière’s groove and the base of segment 4 of the liver are identified as anatomical landmarks. After these landmarks have been confirmed, the Critical View of Safety is attempted by making a dorsal serosal incision in Calot’s triangle lateral to the line connecting these landmarks. If the gallbladder is adherent to adjacent organs, such as the duodenum or transverse colon, if Rouvière’s groove and the base of segment 4 cannot be identified even after decompression by aspirating the contents of the gallbladder, or if significant fibrosis is present around the gallbladder neck, subtotal cholecystectomy is performed to avoid injury to adjacent organs or the bile duct.
(4) Using an ultrasonic coagulation and cutting device, the gallbladder wall is dissected from the neck toward the fundus on the abdominal side, while preserving the wall on the hepatic side (Figure 3a). All stones are removed from the gallbladder (Figure 3b). This creates a cup-shaped remnant around the gallbladder neck, with a residual wall remaining on the fundic side. Because stone recurrence in the remnant gallbladder is a recognized concern after subtotal cholecystectomy, efforts are made to minimize the size of the remnant gallbladder (Figure 3c). Intraoperative cholangiography through the cystic duct orifice is often used to visualize the biliary anatomy and identify retained stones. If stones in the gallbladder neck or cystic duct cannot be completely removed, conversion to open surgery is considered due to the risk of postoperative complications.
(5) The cystic duct orifice is identified within the gallbladder lumen (Figure 3d). When suture closure is selected, the cystic duct orifice is closed at this site using absorbable suture material (Figure 3e,f). The mucosa on the hepatic side of the remnant gallbladder is then ablated (Figure 3g). Closed-suction drains are placed in the right subphrenic space and within the remnant gallbladder cavity (Figure 4).

### 2.4. Definitions and Perioperative Management

Intraoperative bile flow from the cystic duct orifice (IBF) was defined as visible bile outflow from the cystic duct orifice observed within the remnant gallbladder lumen during surgery. IBF is considered an important intraoperative finding because it could influence the surgeon’s decision regarding suture closure of the cystic duct orifice.

Postoperative bile leakage was defined as bilious drainage from the right subphrenic drain after surgery. If bile leakage was evident on direct visual inspection, it was diagnosed clinically. If the bilious nature of the drainage was suspected but not visually obvious, the drain fluid was sent for laboratory analysis; bile leakage was diagnosed when the bilirubin concentration in the drain fluid was at least three times the serum bilirubin concentration, as defined previously by the International Study Group of Liver Surgery [17].

Postoperative drain management was based on the characteristics and volume of the drainage fluid, the patient’s clinical conditions, and postoperative blood test results. The right subphrenic drain was removed when the drainage fluid was clearly non-bilious on direct visual inspection, the drainage volume was low, the patient’s condition was stable, and no elevation in hepatobiliary enzymes or serum bilirubin was observed. In uncomplicated cases, this drain could be removed within 2 postoperative days.

When persistent high-volume bilious drainage from the right subphrenic drain was observed, endoscopic treatment was considered. In our practice, persistent bilious drainage of approximately >50 mL/day prompted consideration of endoscopic interventions, including biliary drainage, endoscopic stent placement, or sphincterotomy. A second drain was placed within the remnant gallbladder cavity. When this drain formed a controlled fistula and inflammatory markers remained stable, it was gradually withdrawn in a stepwise manner and removed completely after approximately 14 days.

### 2.5. Study Parameters and Group Comparisons

Patient background factors and disease characteristics were compared between the non-suture and suture groups, including age, sex, preoperative biliary drainage or intervention and the surgical diagnosis of cholecystitis and its severity (acute or chronic cholecystitis, status of gangrenous cholecystitis, and concomitant Mirizzi syndrome). Intraoperative and postoperative outcomes were also compared, including the presence of IBF, operative time, conversion to open surgery, drain retention period, postoperative hospital stay, postoperative bile leakage and the need for postoperative endoscopic treatment.

In addition, two exploratory subgroup analyses were performed as hypothesis-generating analyses: (1) a comparison between the non-suture group and suture group among patients with IBF; and (2) a comparison between patients with and without IBF within the non-suture group.

### 2.6. Statistical Methods

Statistical analyses were performed using JMP^®^ Student Edition 18.2.2 (JMP Statistical Discovery LLC, SAS Institute Inc., Cary, NC, USA). Categorical variables were analyzed using Fisher’s exact test, while continuous variables were analyzed using the Wilcoxon rank-sum test. Continuous variables were presented as median [interquartile range], and categorical variables as number (%). Effect size estimates with 95% confidence intervals were calculated for the primary comparisons wherever feasible. All tests were two-sided, and *p*-values < 0.05 were considered statistically significant.

### 2.7. Ethics Review

This study was approved by the Research Ethics Committee for Life Science and Medical Research, Tohoku Medical and Pharmaceutical University (2025-2-053).

## 3. Results

No significant differences were observed in baseline patient characteristics, including age, sex, or overall surgical diagnosis of cholecystitis. Although the findings were descriptive, the non-suture group tended to include patients with more severe inflammatory disease, with a higher number of patients with acute cholecystitis and gangrenous cholecystitis, as well as one patient with perforated cholecystitis and one with Mirizzi syndrome. Preoperative biliary drainage or intervention was performed only in the non-suture group, including endoscopic naso-gallbladder drainage (ENGBD) in one patient, endoscopic retrograde biliary drainage (ERBD) in one, and endoscopic papillary balloon dilation (EPBD) in one, whereas no patient in the suture group required preoperative biliary drainage or intervention (Table 1).

A comparative analysis of intraoperative and postoperative outcomes was performed between the non-suture group and the suture group (Table 2). IBF was significantly more frequent in the suture group. One patient in the non-suture group, who had acute cholecystitis with Mirizzi syndrome, required conversion to open surgery because manual removal of an impacted bile duct stone was necessary after division of the gallbladder. The median operative time tended to be shorter in the non-suture group than in the suture group, although the difference was not statistically significant. No significant differences were observed between the groups in terms of drain retention period, postoperative hospital stay, postoperative bile leakage, or the need for postoperative endoscopic treatment. Of the 12 patients diagnosed with postoperative bile leakage, 6 were diagnosed by direct visual inspection of the drain fluid, whereas the remaining 6 were diagnosed based on laboratory analysis of the drain fluid. No reoperations, deaths or readmissions occurred in either group.

As an exploratory subgroup analysis, intraoperative and postoperative outcomes were compared between the non-suture and suture groups among patients with IBF (Table 3). IBF was observed in 10 patients, including 5 in the non-suture group and 5 in the suture group. The median operative time tended to be shorter in the non-suture group than in the suture group. Postoperative bile leakage and postoperative endoscopic treatment were numerically more frequent in the non-suture group, although these differences were not statistically significant.

As an exploratory subgroup analysis, postoperative outcomes were compared between patients with and without IBF within the non-suture group (Table 4). No significant differences were observed in operative time, drain retention period, postoperative hospital stay, or postoperative bile leakage. However, postoperative endoscopic treatment was numerically more frequent in patients with IBF than in those without IBF, although these differences were not statistically significant.

## 4. Discussion

In the present study, we focused specifically on fenestrating LSC and compared perioperative outcomes between patients with and without suture closure of the cystic duct orifice. Postoperative bile leakage occurred in 40.7% of the non-suture group and 14.3% of the suture group, while postoperative endoscopic treatment was required in 25.9% and 14.3%, respectively. Although these differences were not statistically significant, the pattern of the results is noteworthy. In addition, the non-suture group appeared to include patients with more severe inflammatory disease, including more acute and gangrenous cholecystitis, as well as perforation, Mirizzi syndrome, and preoperative biliary drainage or intervention. This suggests that the decision not to suture may have been influenced not only by technical considerations but also by disease severity. Accordingly, these findings should not be interpreted as evidence of equivalence or non-inferiority, but rather as descriptive and hypothesis-generating observations in a small retrospective cohort.

The recent literature comparing fenestrating and reconstituting subtotal cholecystectomy is helpful in interpreting our findings. Recent systematic reviews and meta-analyses have generally suggested that fenestrating subtotal cholecystectomy is associated with a higher incidence of postoperative bile leakage than the reconstituting subtotal cholecystectomy. For example, Ravendran et al. [8] reported bile leakage in 20.8% of fenestrating cases and 12.3% of reconstituting cases; the same review also reported postoperative endoscopic retrograde cholangiopancreatography (ERCP) in 16.1% and 13.2%, respectively. Similarly, Aloraini et al. [14] reported a higher bile leakage rate with the fenestrating method than with the reconstituting method (23.1% vs 10.0%), although the difference was not statistically significant. Ahmed et al. [15] also found a significantly longer postoperative hospital stay with fenestrating LSC, while bile leakage, postoperative ERCP, and reoperation rates were not significantly different between techniques. Against this background, the present findings do not conflict with the recent literature. Rather, the higher rates of postoperative bile leakage and postoperative endoscopic treatment in our non-suture group are in line with the general tendency seen in previous reports, although our sample was too small to determine whether these differences were reliable.

A recent retrospective study by Gross et al. [13] is particularly relevant because it addressed cystic duct closure within fenestrating subtotal cholecystectomy rather than only the broader fenestrating-versus-reconstituting comparison. They reported that postoperative bile leakage decreased from 24.1% to 6.0% when cystic duct closure was achieved in the fenestrating method. More recently, Johnson et al. [18] reported in a large contemporary cohort that fenestrating subtotal cholecystectomy was associated with more bile leak than reconstituting subtotal cholecystectomy (22.0% vs 6.9%) and with more postoperative ERCP (36.3% vs 19.4%). A similar trend was observed in our study: the non-suture group had more postoperative bile leakage and required more postoperative endoscopic treatment than the suture group. Therefore, our study does not show that omission of cystic duct orifice closure is equivalent to suture closure. Although our findings are broadly in line with recent literature favoring suture closure, firm conclusions cannot be drawn because of the small sample size and the marked imbalance between groups.

Several factors may explain why our absolute rates differed from those reported in some published series. First, the decision to perform suture closure in our cohort was not protocolized and was left to the operating surgeon according to intraoperative findings. The suture group had a significantly higher frequency of IBF, indicating that visible bile flow itself strongly influenced the decision to attempt closure. By contrast, the non-suture group appeared to include more severe inflammatory disease and more difficult local conditions. These imbalances suggest substantial confounding by indication. The two groups were likely characterized by different kinds of difficulty: the suture group by obvious bile flow from the cystic duct orifice, and the non-suture group by severe inflammation, tissue friability, and difficult anatomy. Second, postoperative management may have differed from that in other published cohorts. In our institution, fenestrating LSC was accompanied by active drain placement, close monitoring of bilious drainage, and early endoscopic intervention when necessary. This strategy may have reduced the need for reoperation, because postoperative bile leakage could often be managed with drainage and endoscopic treatment rather than surgery.

This interpretation is also supported by comparison with pooled outcomes after subtotal cholecystectomy. Elshaer et al. [2] reported retained stones in 3.1%, postoperative ERCP in 4.1%, reoperation in 1.8%, and postoperative bile leakage in 18.0% after subtotal cholecystectomy overall. More recently, a systematic review and meta-analysis by Nadeem et al. [19] reported that postoperative bile leakage occurred in 13.5%, retained stones in 6.1%, postoperative ERCP in 16.2%, readmission in 17.8%, reoperation in 6.3%, and mortality in 0.8%. In our cohort, postoperative bile leakage and endoscopic treatment rates in the suture group were within or near the ranges reported previously, whereas the non-suture group showed a higher postoperative bile leakage rate and a somewhat higher endoscopic treatment rate. However, no reoperations, deaths, or readmissions occurred in either group. This may suggest that close drain monitoring and timely endoscopic drainage or intervention may have reduced progression to more severe postoperative complications. Nevertheless, cross-study comparisons should be interpreted cautiously because definitions of bile leakage, indications for endoscopic intervention, patient backgrounds, and disease severity may differ among studies.

The exploratory subgroup analyses in our study should also be interpreted carefully, but they might still provide useful clinical signals. Among patients with IBF, the median operative time was 94 min in the non-suture group and 172 min in the suture group, while postoperative bile leakage and postoperative endoscopic treatment each occurred in 60.0% versus 20.0%, respectively. These findings suggest a possible trade-off: omission of intracorporeal suturing may shorten operative time in technically difficult cases, but may also increase the burden of postoperative management. Similarly, within the non-suture group, postoperative bile leakage occurred in 60.0% of IBF-present patients versus 36.4% of IBF-absent patients, while postoperative endoscopic treatment was required in 60.0% versus 18.2%, respectively. Although these subgroup comparisons are based on very small numbers and should not be overinterpreted, they suggest that IBF may be a clinically relevant intraoperative marker of postoperative management burden, particularly when cystic duct orifice closure is omitted. Therefore, these trends may be regarded as signals that need confirmation in larger cohorts rather than as a basis for practice recommendations.

The findings of the present study should also be interpreted in the context of the long-term consequences of subtotal cholecystectomy, although such outcomes were not evaluated here. Long-term remnant-related morbidity has been reported in several series. Kohga et al. [20] showed that remnant gallbladder size on postoperative magnetic resonance cholangiography was associated with later complications related to retained stones. Slater et al. [21], in a 13-year series of LSC, reported that 32% of patients underwent re-investigation for upper abdominal symptoms and the interval to re-intervention ranged from 21 days to 124 months after surgery. These studies indicate that remnant-related events are clinically relevant and may require later intervention. By contrast, robust cohort data defining the incidence of remnant gallbladder cancer after subtotal cholecystectomy remain limited. These considerations underscore that the present study should be interpreted as addressing only perioperative outcomes, not the long-term remnant-related or oncologic consequences of fenestrating LSC.

This study has several limitations. First, it was a retrospective and single-center study, and the decision to perform suture closure was strongly influenced by intraoperative judgment. Therefore, the suture and non-suture groups were not fully comparable, and the observed differences may have been influenced by case selection. Second, the sample size was small, particularly in the suture group and in the exploratory subgroup analyses, and the study was not powered to detect modest but clinically meaningful differences between the groups. Third, although effect sizes were added for the primary comparisons, the confidence intervals were wide, showing the uncertainty of the observed associations. Fourth, the subgroup analyses were exploratory and particularly unstable because of the very small numbers involved. Fifth, the patients in this study were somewhat older than those in recent reports, in which the mean age of the study populations ranged from approximately 48 to 61 years [13,14,15]. In the present study, the median age was 77 years in the non-suture group and 76 years in the suture group. Consequently, these findings may be difficult to generalize to younger age groups. Advanced age may have influenced intraoperative decision-making, tissue fragility, and the risk of postoperative complications. Finally, our analysis was limited to perioperative outcomes and does not address long-term remnant-related complications. For these reasons, the present findings should be considered hypothesis-generating.

## 5. Conclusions

In this retrospective single-institution study, no statistically significant differences in perioperative outcomes were observed between the non-suture and suture groups during fenestrating LSC. However, the non-suture group showed a trend toward higher rates of postoperative bile leakage and endoscopic treatment. Given the small sample size and the potential influence of case selection and confounding, these hypothesis-generating findings should be interpreted with caution.

## Figures and Tables

**Table 1:** Comparison of patient background factors and disease characteristics between the non-suture and suture groups.

	Non-Suture Group (*n* = 27)	Suture Group (*n* = 7)	*p*-Value
Age, years Median [IQR]	77 [70–83]	76 [71–81]	0.765
Male, N (%)	18 (66.7)	6 (85.7)	0.644
Surgical diagnosis of cholecystitis, N (%)	26 (96.3)	7 (100)	1.0
Acute cholecystitis With Mirizzi syndrome Perforated Gangrenous Chronic cholecystitis	23 1 1 14 4	4 0 0 1 3	
Preoperative biliary drainage or intervention, N (%) ENGBD ERBD EPBD	3 (11.1) 1 1 1	0(0)	1.0

Data are presented as median [IQR] for continuous variables and number (%) for categorical variables. IQR: interquartile range; ENGBD, endoscopic naso-gallbladder drainage; ERBD, endoscopic retrograde biliary drainage; EPBD, endoscopic papillary balloon dilation.

**Table 2:** Comparison of intraoperative and postoperative outcomes between the non-suture and suture groups.

	Non-Suture Group (*n* = 27)	Suture Group (*n* = 7)	Relative Risk [95%CI]	*p*-Value
IBF present, N (%)	5 (18.5)	5 (71.4)		0.014
Median operative time, minutes Median [IQR]	115 [95.5–139]	135 [119.5–174.5]		0.074
Median drain retention period, days Median [IQR]	7.5 [3–16]	7.5 [6–13]		0.576
Median postoperative hospital stay, days Median [IQR]	13 [7.5–20.5]	11 [9–13.5]		0.983
Postoperative bile leakage, N (%)	11 (40.7)	1 (14.3)	* 0.351 [0.054–2.28]	0.378
Postoperative endoscopic treatment, N (%)	7 (25.9)	1 (14.3)	* 0.551 [0.081–3.77]	1.0

Data are presented as median [IQR] for continuous variables and number (%) for categorical variables. IQR, interquartile range; CI, confidence interval; IBF, intraoperative bile flow from the cystic duct orifice. * Risk ratio is presented for the suture group relative to the non-suture group.

**Table 3:** Comparison of intraoperative and postoperative outcomes between the non-suture and suture groups in patients with IBF.

	Non-Suture Group (*n* = 5)	Suture Group (*n* = 5)	*p*-Value
Operative time, minutes Median [IQR]	94 [85–115]	172 [125–177]	0.060
Drain retention period, days Median [IQR]	13 [8–14]	11.5 [7.5–17]	0.753
Postoperative hospital stay, days Median [IQR]	18 [15–27]	11 [9–16]	0.462
Postoperative bile leakage, N (%)	3 (60.0)	1 (20.0)	0.524
Postoperative endoscopic treatment, N (%)	3 (60.0)	1 (20.0)	0.524

Data are presented as median [IQR] for continuous variables and number (%) for categorical variables. IQR: interquartile range; IBF, intraoperative bile flow from the cystic duct orifice.

**Table 4:** Comparison between patients with and without IBF in the non-suture groups.

	IBF Present (*n* = 5)	IBF Absent (*n* = 22)	*p*-Value
Operative time, minutes Median [IQR]	94 [85–115]	116.5 [107.5–139.5]	0.365
Drain retention period, days Median [IQR]	13 [8–14]	5 [3–16]	0.430
Postoperative hospital stay, days Median [IQR]	18 [15–27]	11.5 [7–17.5]	0.188
Postoperative bile leakage, N (%)	3 (60.0)	8 (36.4)	0.370
Postoperative endoscopic treatment, N (%)	3 (60.0)	4 (18.2)	0.091

Data are presented as median [IQR] for continuous variables and number (%) for categorical variables. IQR: interquartile range; IBF, intraoperative bile flow from the cystic duct orifice.

## Data Availability

The data supporting the findings of this study are available from the corresponding author upon reasonable request.

## Abbreviations

The following abbreviations are used in this manuscript:

LSC	Laparoscopic subtotal cholecystectomy
IBF	Intraoperative bile flow from the cystic duct orifice
ENGBD	Endoscopic naso-gallbladder drainage
ERBD	Endoscopic retrograde biliary drainage
EPBD	Endoscopic papillary balloon dilation
ERCP	Endoscopic retrograde cholangiopancreatography
